# Restraint stress during neonatal hypoxia‐ischemia alters brain injury following normothermia and hypothermia

**DOI:** 10.14814/phy2.15562

**Published:** 2023-01-12

**Authors:** Julia K. Gundersen, Hemmen Sabir, Thomas R. Wood, Damjan Osredkar, Mari Falck, Else M. Loeberg, Lars Walloe, David A. Menassa, Marianne Thoresen

**Affiliations:** ^1^ Department of Physiology Institute of Basic Medical Sciences, University of Oslo Oslo Norway; ^2^ Department of Neonatology and Pediatric Intensive Care Children's Hospital University of Bonn Bonn Germany; ^3^ Department of Pediatrics University of Washington Medical School Seattle Washington USA; ^4^ Department of Pediatrics Ljubljana University Hospital Ljubljana Slovenia; ^5^ Department of Pediatrics Oslo University Hospital Oslo Norway; ^6^ Department of Pathology Oslo University Hospital Oslo Norway; ^7^ The Queen's College| University of Oxford Oxford UK; ^8^ Translational Health Sciences, St. Michael's Hospital, Bristol Medical School University of Bristol Bristol UK

**Keywords:** hypothermia, hypoxia‐ischemia, neuroprotection, stress, Vannucci‐model

## Abstract

Rodent models of neonatal hypoxic–ischemic (HI) injury require a subset of animals to be immobilized for continuous temperature monitoring during the insult and subsequent treatment. Restrained animals are discarded from the analysis due to the effect of restraint on the brain injury as first demonstrated by Thoresen et al 1996. However, the effects of restraint on responses to hypothermic (HT) post‐insult therapy are not well described. We examine the effects of restraint associated with different probe placements on HI brain injury. We have conducted a meta‐analysis of 23 experiments comparing probe rats (skin *n* = 42, rectal *n* = 35) and free‐moving matched non‐probe controls (*n* = 80) that underwent HI injury (left common carotid artery ligation and 90 min 8% O_2_) at postnatal day 7 (P7), followed by 5 h of NT (37°C) or HT (32°C). On P14, brain regions were analyzed for injury (by neuropathology and area loss), microglial reactivity and brain‐derived neurotrophic factor (BDNF). HI injury was mitigated in NT skin and rectal probe rats, with greater neuroprotection among the rectal probe rats. Following HT, the skin probe rats maintained the restraint‐associated neuroprotection, while brain injury was significantly exacerbated among the rectal probe rats. Microglial reactivity strongly correlated with the acquired injury, with no detectable difference between the groups. Likewise, we observed no differences in BDNF signal intensity. Our findings suggest a biphasic neuroprotection from restraint stress, which becomes detrimental in combination with HT and the presumed discomfort from the rectal probe. This finding is useful in highlighting unforeseen effects of common experimental designs or routine clinical management.

## Graphical abstract

Rodent models of hypoxic–ischemic brain injury require some animals to be restrained for continuous physiological recordings during the experiment. Compared to free‐moving controls, restrained rats achieve less brain injury following post‐insult normothermia, whereas post‐insult hypothermia exacerbate the injury in rats carrying a rectal probe. Our findings suggest a protective effect from restraint during physiological temperature, which becomes harmful when combined with hypothermia and the additional discomfort from the rectal probe.

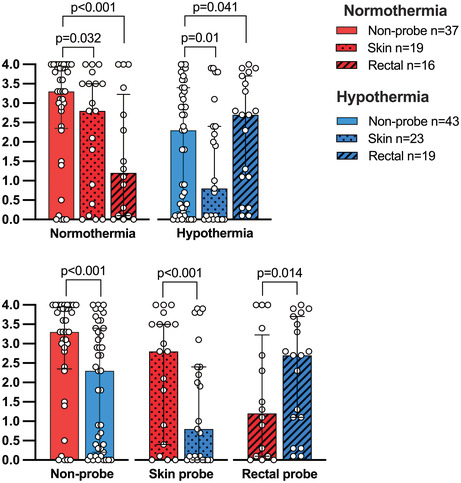

## 1 BACKGROUND

Perinatal hypoxic–ischemic (HI) brain injury is a major cause of neonatal mortality and neurodevelopmental morbidity (Kurinczuk et al., 2010). The Vannucci model (Vannucci & Back, 2022; Vannucci & Vannucci, 1997, 2005) in postnatal day 7 rats (P7) (Semple et al., 2013; Yager et al., 1996) is a widely‐used experimental model of neonatal HI injury and is based on the Levine model (Levine, 1960) in adult rats. The Vannucci model has become the animal model of choice in exploring therapeutic treatments for HI injury (Vannucci & Vannucci, 2005), such as hypothermia (HT), with which our group has 30 years of experience (Thoresen, Bagenholm, et al., 1996). Notwithstanding validation across numerous labs, the Vannucci model is inherently variable in both injury and neuroprotection from HT (Wood et al., 2020), indicating unknown mediating mechanisms and influencers of injury response.

Temperature control during and after HI injury is critical to reliably produce consistent brain injury (Busto et al., 1987, 1989) and to draw robust conclusions on effective neuroprotective interventions, such as HT (Galinsky et al., 2017). For this reason, a subset of experimental animals is immobilized to allow for continuous core temperature monitoring during hypoxia and subsequent treatment. These so‐called “probe rats” are subjected to potential additional stress and discomfort of restraint and are therefore usually discarded from the final analysis. Twenty‐six years ago, we demonstrated reduced brain injury in normothermic probe rats as compared to rats not carrying a rectal thermometer probe (Thoresen, Bågenholm, et al., 1996). It was hypothesized that the stress of being restrained during the experiment ameliorated the injury, but this unexpected finding needed validation in a different lab. The effects of stress on responses to therapies such as post‐insult HT remain widely unexplored in both the Vannucci model and in the clinical setting. Patients often require some immobilization during neonatal intensive care for continuous monitoring, drug infusion and mechanical ventilation, and experience a series of invasive and often painful procedures. Pain‐related stress has been demonstrated to be harmful to the immature human brain (Chau et al., 2019; Ranger et al., 2013, 2015) and to reduce the effectiveness of HT following HI injury in newborn pigs (Thoresen et al., 2001). For this reason, patients undergoing HT are usually sedated and mechanically ventilated; however, this is unfeasible to do in an 11 gram rat pup.

Restraint is a commonly used experimental stressor in rodent models (Paré & Glavin, 1986; Pitman et al., 1988). Stress is linked to mitochondrial impairment (Picard & McEwen, 2018), blood–brain‐barrier disruption (Welcome & Mastorakis, 2020) and endothelial dysfunction (Carda et al., 2015), all potential mediators of HI injury. Stress may also alter injury responses through neurotrophins, such as brain‐derived neurotrophic factor (BDNF), a peptide growth factor ubiquitously expressed in varying concentrations in the central nervous system (Notaras & van den Buuse, 2020). BDNF is recognized as a potential neuroprotective agent (Chen et al., 2013), possibly acting through the inhibition of apoptosis (Hee Han et al., 2000). Elevated levels of BDNF are detected up to 30 days following neonatal HI injury (Diaz et al., 2017), and pre‐treatment with intraventricular BDNF ameliorated HI brain injury and improved spatial learning and memory in P7 rats (Almli et al., 2000).

This study aims to examine the effect of immobilization associated with rectal versus superficial skin probe placement during HI injury and 5 h post‐insult treatment with NT or HT.

## 2 METHODS

### 2.1 Animals

All experiments were approved by the University of Oslo's Animal Ethics Research Committee and performed by individuals holding an approved license according to the Animal act 1986, FOTS ID: 4344. The experiments included in our analysis were conducted on P7 Wistar rats imported from Charles River laboratories, Sulzfeld, Germany. The rats were kept with their dam in an animal facility with 12:12 h day:night cycle, at room temperature of 21°C with food and water ad libitum. To achieve a sufficient sample size of probe rats, we collected data from 23 experiments previously published and conducted in the Oslo laboratory between 2013 and 2016 (Wood et al., 2020). Thirty‐four percent of the litters were crossfostered (Gundersen, Menassa, et al., 2021). Each experiment was conducted on a set of five or six litters of ten pups each exposed to hypoxia in one large chamber with 60 individual partitions. Among these, four pups were arbitrarily chosen to carry a skin or rectal thermometer probe (rectal probe: SI‐451 Mouse Temperature Probe, cwe‐inc.com, 1 mm diameter) during the experimental period (6.5 h). The probe rats were immobilized against the chamber floor with adhesive tape to not entangle in the cables attached to the probe. Among all non‐probe rats in these experiments (*n* = 642), 37 died (6% mortality, *n* = 20 during ligation, *n* = 10 during insult, *n* = 7 post‐insult). Among all probe rats, four rectal probe rats (*n* = 2 unsuccessful ligation, *n* = 2 not stated) and two skin probe rats (*n* = 1 unsuccessful ligation, *n* = 1 died during hypoxia) were excluded from analysis. In total, 42 rats carrying a skin probe and 35 carrying a rectal probe were included. For each probe rat, a representative “non‐probe” rat not immobilized or carrying a thermometer probe was selected (*n* = 80) based on the median hemispheric area loss within the same litter and treatment group, and preferably same sex. We confirmed a representative selection of the non‐probe rats (*n* = 80) by comparing the distribution of hemispheric area loss in all non‐probe rats of the 23 experiments analyzed (*n* = 642) and in all non‐probe rats in the selected litters with at least one designated probe rat (*n* = 378).

### 2.2 Study design

All experiments were performed on P7 using the Vannucci model to produce moderate HI injury. In brief, all pups underwent unilateral ligation of the left common carotid artery under anesthesia (3% isoflurane in a 2:1 gas mixture of NO_2_/O_2_), followed by a recovery period for at least 30 min. Afterwards, pups were placed in a hypoxia‐chamber and exposed to 8% O_2_ for 90 min at 36.0°C rectal temperature. During hypoxia, levels of O_2_ and CO_2_ within the chamber were continuously monitored. The core temperature was registered from the designated rats carrying a rectal thermometer probe, which correlates within 0.1°C with the brain temperature (Thoresen, Bagenholm, et al., 1996). A servo‐controlled water‐filled system (Criticool, MTRE, Yavne, Israel) maintained the rectal temperature within ±0.2°C, thereby ensuring all experimental animals maintained the same environmental temperature as the rectal probe rat. Rats carrying skin thermometer probes were also restrained and used to monitor the corresponding skin temperature. Rats not carrying a probe were free to move spontaneously within the confined space (8 × 5 × 3 cm^3^) in a partition, while the probe rats required adhesive tape to secure the probe, which in practice immobilized them. For the analysis, the continuous temperature recordings were sampled at baseline, every 15 min during the insult and then every 30 min during the treatment.

All pups were randomized by litter, weight, and sex to 5 h treatment of either normothermia (NT; 37°C ± 0.2°C rectal temperature), or HT (32°C ± 0.2°C rectal temperature). Pups were transferred to their allocated pre‐heated or pre‐cooled treatment chamber immediately after hypoxia. After 5 h of treatment, the HT group were rewarmed at 1°C every 15 min until the probe animal recorded a rectal temperature of 34.5 ± 0.5°C, after which all pups were returned to their dam.

### 2.3 Tissue sampling

On P14, all pups underwent trans‐cardiac perfusion with 10% phosphate‐buffered formaldehyde (0.1 M) under isoflurane anesthesia. The brain was extracted and kept in formaldehyde for 4 days until further processing. Six coronal 3 mm blocks, numbered 1 (frontal) to 6 (caudal), were cut through the brain (ASI Instruments Inc.) and embedded in paraffin. Slices (6 μm) were cut from block 3 (basal ganglia) and block 4 (hippocampus, thalamus) for histological staining with hematoxylin and eosin (H&E). Only block 4 was used for immunohistochemistry.

### 2.4 Immunohistochemistry

Sections were deparaffinized at 60°C for 1 h followed by incubation in 100% xylene (15 min) and graded rehydration at 5‐min intervals in absolute ethanol (10 min), 96% ethanol, 80% ethanol, 70% ethanol and in distilled water. The sections were washed in a solution of 0.1% tween‐20‐phosphate buffer saline (0.1% PBS‐T). Afterwards, antigen retrieval was performed by boiling the section in 10 mM citrate buffer (pH = 6.0) at 96°C for 25 min.

For brightfield immunohistochemistry, dual enzyme block (Dako, S2003, Agilent) was applied for 10 min to block endogenous peroxidases and phosphatases and all sections were then incubated for 1 h in 10% horse serum in 0.2% PBS‐T. Primary antibodies against NeuN (mouse anti‐rat; 1:1500, Abcam ab‐104,224) and Iba‐1 (rabbit anti‐rat; 1:750, Wako 013–27,691) were applied for 48 h in 10% horse serum +5% bovine serum albumin (BSA) in 0.2% PBS‐T. Following incubation, primary antibodies were washed off in 0.1% PBS‐T. Secondary antibodies were applied (horse‐anti‐rabbit HRP (brown) + horse‐anti‐rabbit AP (magenta)) using the Immpress Duet Double Staining Polymer kit (MP‐7724, Vector Laboratories). Visualization of epitopes was achieved with DAB and AP chromogens applied sequentially. Sections were counterstained in non‐diluted hematoxylin for 45 s and washed off in distilled water. Lastly, sections were dehydrated in 70% ethanol, 80% ethanol, 96% ethanol and cleared in absolute ethanol and 100% xylene before they were mounted with permanent mounting medium (DPX).

For immunofluorescence, after antigen retrieval and washing all sections were incubated for 1 h in 10% goat serum in 0.2% PBS‐T. Afterwards, a BDNF primary antibody (rabbit anti‐rat, 1:3000, Abcam ab213323) in a solution of 10% goat serum (GS) + 5% BSA in 0.2% PBS‐T was applied for 48 h incubation period. Following washing, an Alexa fluor plus 488 (goat‐anti‐rabbit, 1:1000, Invitrogen A32731) secondary antibody was applied in 10% GS + 5% BSA + 0.2% PBS‐T for 1 h. Lastly, all sections were washed and mounted with DAPI mounting medium.

### 2.5 Histological assessment

All sections were scanned as high‐resolution micrographs with a Zeiss Axioscan Z1 (pixel resolution: 0.220 × 0.220 μm^2^, objective: plan/apochromat 20x/0.8 M27). The histological assessment was performed by investigators blinded to the treatment group and probe‐status.

Histopathology is the gold standard for assessing HI injury. The regional injury was assessed in the hippocampus (CA1‐CA4 and gyrus dentatus), cortex, thalamus and basal ganglia using a validated 9‐point (0.0–4.0) histopathology score as previously described (Gundersen, Menassa, et al., 2021; Thoresen, Bagenholm, et al., 1996). The grade was decided by regional morphological changes of necrotic neurons, complete or incomplete infarction and distribution of injury and tissue disintegration. The global pathology score was calculated by averaging the four regional scores.

Gross hemispheric injury was assessed in the same sections analyzed for regional pathology score using ImageJ (v.1.46r, National Institutes of Health, Bethesda, MD, USA). The relative area loss of the injured hemisphere was measured by pixel intensity thresholding and calculated by the formula: (1‐left area/right area)*100 in sections representing block 3 and 4, of which the average value was calculated (Wood et al., 2020).

Based on preliminary results, microglial reactivity was assessed in NeuN‐Iba1 stained sections in the cornu ammoni of the hippocampus and the thalamus by measuring the nearest neighbor distance (NND). The analysis was semi‐automated using batch‐processing in ImageJ. Images were color deconvolved and pixel intensity thresholded, followed by the *“analyze particles”‐*function which selected cells within a size range of 40–500 μm^2^. NND was calculated using the NND‐macro available online (Yuxiong, 2016).

BDNF fluorescence intensity was measured (Shihan et al., 2021) in the hippocampus, cortex and thalamus. In brief, images in .czi format were opened in ImageJ as hyperstack with colorized split channels. In the hippocampus, the region of interest (i.e. hippocampal cell layer) was drawn and the mean intensity was measured with the “*measure”* function. In the cortex, the region of interest was selected to in the medial‐most cortical area. Neurons were first identified using a dynamic range threshold macro, selected using “*analyze particles”* for size 15–250 μm^2^ and measured. Background autofluorescence was accounted for by dividing the region of interest signal intensity by the overall signal intensity of the hippocampus or cortex: (cell signal intensity/overall intensity)*100.

### 2.6 Statistical analysis

Statistical analyses were performed in SPSS Statistics (v.28.0.), STATA (v.17.0, StataCorp LLC.) and GraphPad Prism 9 (GraphPad Software). We applied non‐parametric statistics as the data were not normally distributed. To verify representative selection of the non‐probe controls, we employed the Kolmogorov–Smirnov two‐sample test to compare distribution of the hemispheric area loss between all non‐probe rats of the 23 experiments analyzed (*n* = 642), all non‐probe rats in the selected litters (*n* = 378) and the representative selection of non‐probe controls (*n* = 80). The Wilcoxon Mann–Whitney two‐sample test was used to compare observed medians and distributions between treatment groups for hemispheric area loss, microglial reactivity and BDNF signal intensity. The Wilcoxon van‐Elteren test (van Elteren, 1960), a stratification of the Wilcoxon Mann–Whitney, was used to detect differences in regional pathology score, with each region set as a strata. Differences between the sexes and between crossfostered and non‐crossfostered animals were further investigated with Wilcoxon Mann–Whitney with Bonferroni correction for multiple comparisons (*n* = 6 comparisons, *p*‐value for significance = 0.05/6 = 0.008). Kruskal‐Wallis one‐way analysis of variance was performed to detect statistical differences in weight among all groups. Correlations were examined using Kendall's Tau‐b. All figures were created in GraphPad. All values are presented as medians with interquartile range (IQR) and all other *p*‐values <0.05 were considered statistically significant.

## 3 RESULTS

A total of 157 rats were included in the final analysis (*n* = 80 non‐probe controls, *n* = 42 skin probe rats and *n* = 35 rectal probe rats). Weight at P6 was similar in non‐probe controls (11.2 g; 10.3–12.2), skin probe rats (11.0 g; 9.7–12.7) and rectal probe rats (11.6 g; 10.6–12.6). At P14, weight remained comparable between non‐probe controls (22.9 g; 19.9–24.9), skin probe rats (22.8 g; 19.8–24.6) and rectal probe rats (21.3 g; 19.0–25.6), indicating that the rectal thermometer probe did not affect bowel movement after removal.

In the NT‐group, injury distribution was comparable between the selected non‐probe controls (*n* = 37, 52.7%; 40.0–58.8) and all non‐probe rats in the selected experiments (*n* = 318, 50.9%; 15.6–60.3, *p* = 0.468) and selected litters which contained at least one probe rat (*n* = 186, 50.2%; 14.2–60.3, *p* = 0.587). Likewise in HT, the injury distribution was comparable between non‐probe controls (*n* = 43, 41.9%; 6.4–52.4) and all non‐probe rats of the experiments (*n* = 324, 41.4%; 8.9–54.9, *p* = 0.469) and selected litters (*n* = 192, 45.6%; 8.2–55.2, *p* = 0.702).

### 3.1 Temperature recordings

**FIGURE 1:**
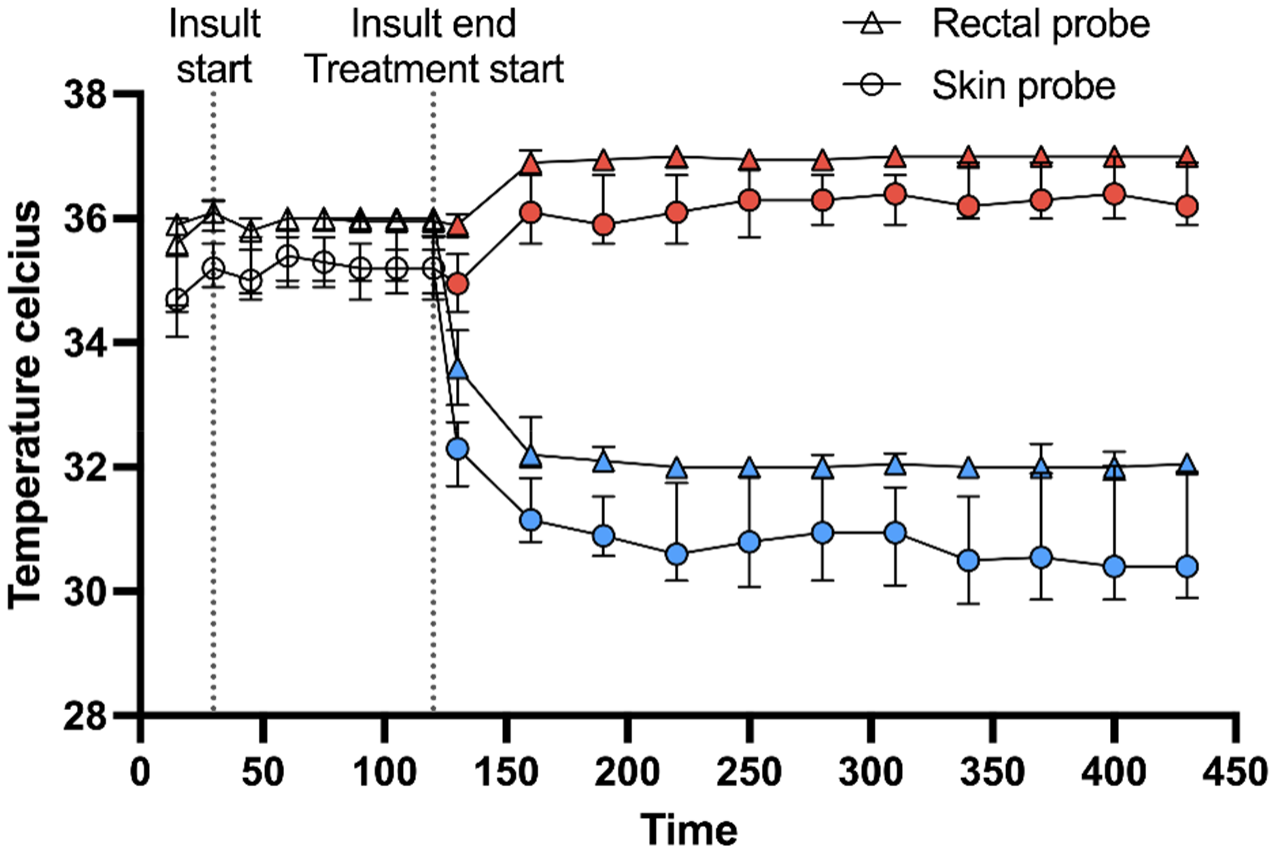
Skin and rectal temperature. Continuous temperature recordings from the skin or rectal probe rats were sampled at 15 min at baseline, every 15 min during the hypoxia‐ischemia insult (total 90 min) and every 30 min during the treatment (5 h). Figure shows the median (IQR) at each timepoint. Throughout the insult duration, the difference in temperature recorded between rectal and skin probe rats within the same experiment and treatment was 0.9°C. During treatment, this difference increased to 1.6°C for HT, but remained at 0.9°C for NT. This end‐of‐treatment temperature difference was not significantly correlated with global pathology score (*p* > 0.50).

The baseline rectal and skin temperatures recorded (Figure 1) were similar in both treatment groups. The median difference in the recorded temperature of the skin and rectal probe within the same treatment group and experiment was 0.9°C at baseline and 0.8°C during hypoxia. At the end of the 5 h treatment period, this difference was maintained at 0.8°C in the NT‐group, while the HT temperature difference between skin and rectal probes increased to 1.9°C. This end‐of‐treatment difference did not correlate with the global pathology score of either the skin probe rats (Kendall's tau‐b − 0.20, *p* = 0.13) or rectal probe rats (Kendall's tau‐b 0.11, *p* = 0.39).

### 3.2 Regional histopathology

**FIGURE 2:**
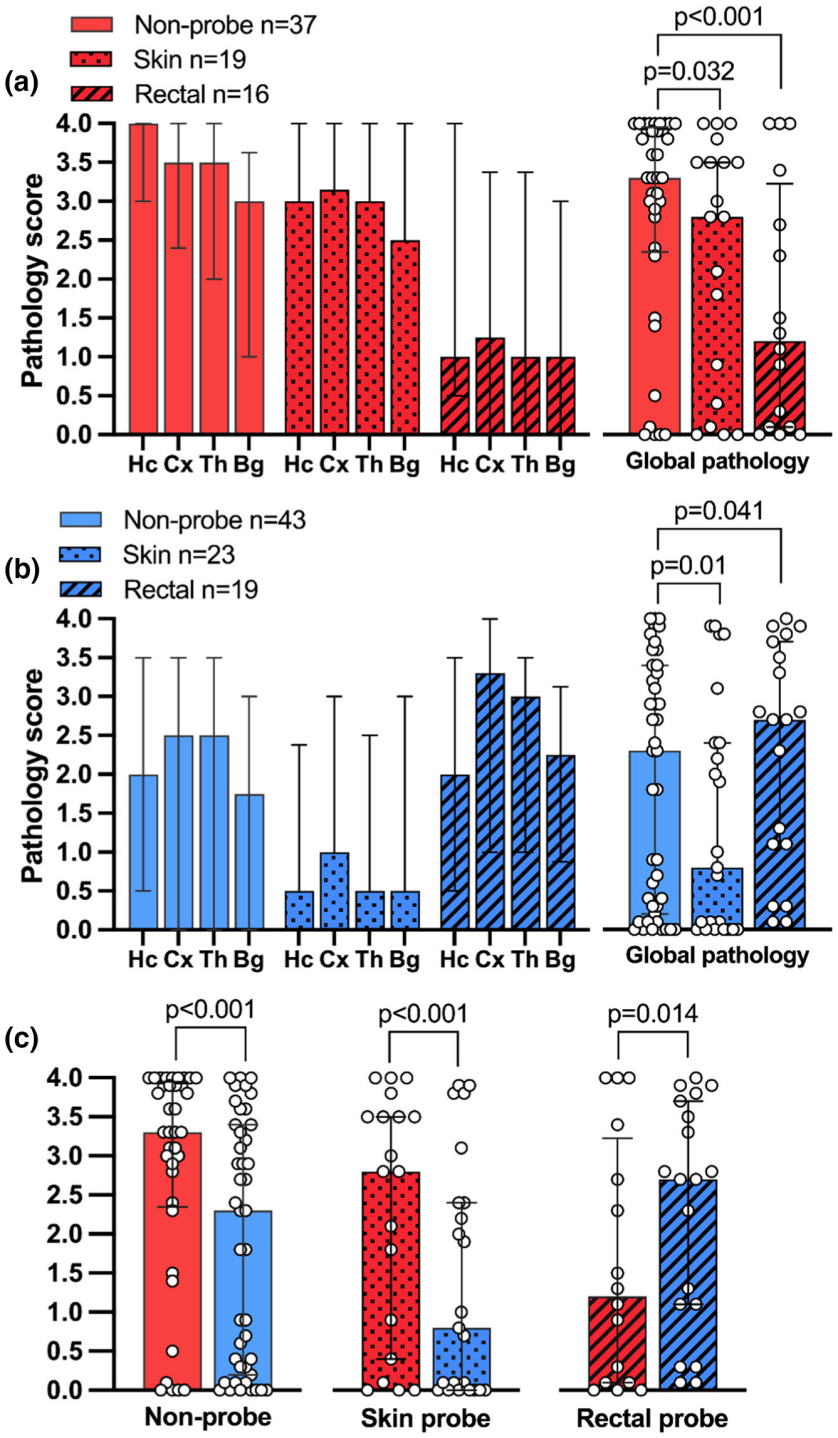
Regional histopathology. Bar graph (median at bar line, IQR) of regional pathology score (Hc = hippocampus, cx = cortex, Th = thalamus, Bg = basal ganglia) and global pathology score of the left hemisphere in non‐probe controls, skin probes and rectal probes, stratified by treatment: (a) normothermia and (b) hypothermia. Figure (c) shows the same global pathology score, now stratified by probe status to better visualize neuroprotection from HT (c). The statistics presented use the two‐sample Wilcoxon van‐Elteren test (van Elteren, 1960).

NT non‐probe controls achieved a median (IQR) global pathology score of 3.31 (2.38–3.88). In comparison, the global injury was significantly mitigated among NT skin probe rats (2.81; 0.48–3.50, *p* = 0.032) and still greater mitigated in NT rectal probe rats (1.22; 0.13–3.03, *p* < 0.001) (Figure 2). In the HT treatment arm, the non‐probe controls acquired a global pathology score of 2.25 (0.19–3.38) while the HT skin probe rats scored significantly less (0.75; 0.0–2.38, *p* = 0.01). In contrast, the injury acquired by the HT rectal probe rats was exacerbated (2.69; 1.13–3.69) compared to the HT non‐probe controls (*p* = 0.041). The therapeutic effect of HT was neuroprotective among non‐probe controls (NT vs HT, *p* < 0.001) and skin probe rats (*p* < 0.001) (Figure 2). The deleterious effect of HT in the rectal probe rats was also statistically significant (NT vs. HT, *p* = 0.014).

We observed no significant differences between the sexes in global or regional pathology score in either of the groups, and there were no difference between crossfostered and non‐crossfostered rats (data not shown).

### 3.3 Hemispheric area loss

**FIGURE 3:**
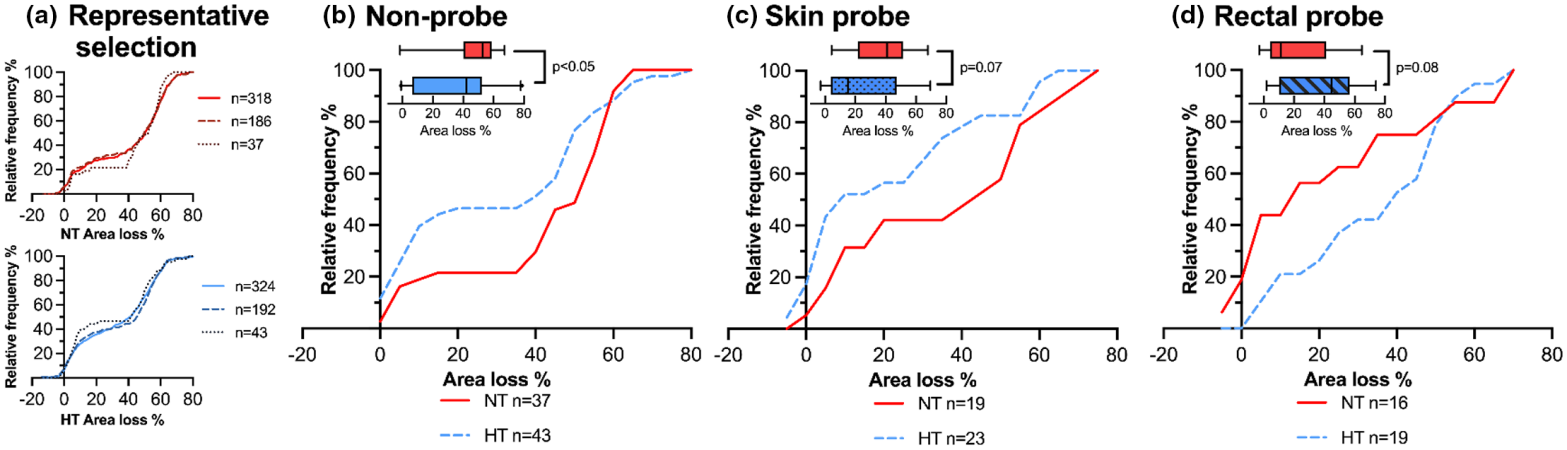
Hemispheric area loss. Cumulative plots with corresponding box‐plots (median, IQR, 2.5–97.5 percentile) of left hemispheric area loss stratified by treatment (NT red, HT blue) and probe condition. We confirmed a representative selection (a) of the non‐probe controls (NT *n* = 37, HT *n* = 43) by comparing distribution of injury in all non‐probe rats in the selected experiments (NT *n* = 318, HT *n* = 324) and all non‐probe rats in the litters which contained at least one probe rat (NT *n* = 186, HT *n* = 192), which was not statistically different. Subfigures b, c, and d. show the hemispheric area loss stratified by treatment in non‐probe controls (b), skin probe rats (c) and rectal probe rats (d). The statistics presented use the two‐sample Wilcoxon Mann–Whitney test.

Hemispheric area loss correlated with global pathology score (Kendall's tau‐b 0.82, *p* < 0.001). With hemispheric area loss, we were unable to demonstrate statistically significant differences between non‐probe controls and skin‐probe rats. HT was neuroprotective among non‐probe controls (Figure 3, NT: 52.7%; 40.0–58.8 vs. HT: 41.9%; 6.4–52.4, *p* = 0.048). In skin probe rats, HT neuroprotection was noted but did not reach significance: (44.9%; 10.2–56.8 vs. HT: 11.2%; 4.1–41.7, *p* = 0.065). The area loss in NT rectal probe rats (15.2%; 3.9–47.1) was dramatically reduced in comparison to NT non‐probe controls (*p* = 0.016). HT exacerbated the injury in rectal probe rats (NT vs. HT, *p* = 0.06), who acquired an area loss of 41.0% (21.8–51.3; Figure 3).

### 3.4 Microglial reactivity

**FIGURE 4:**
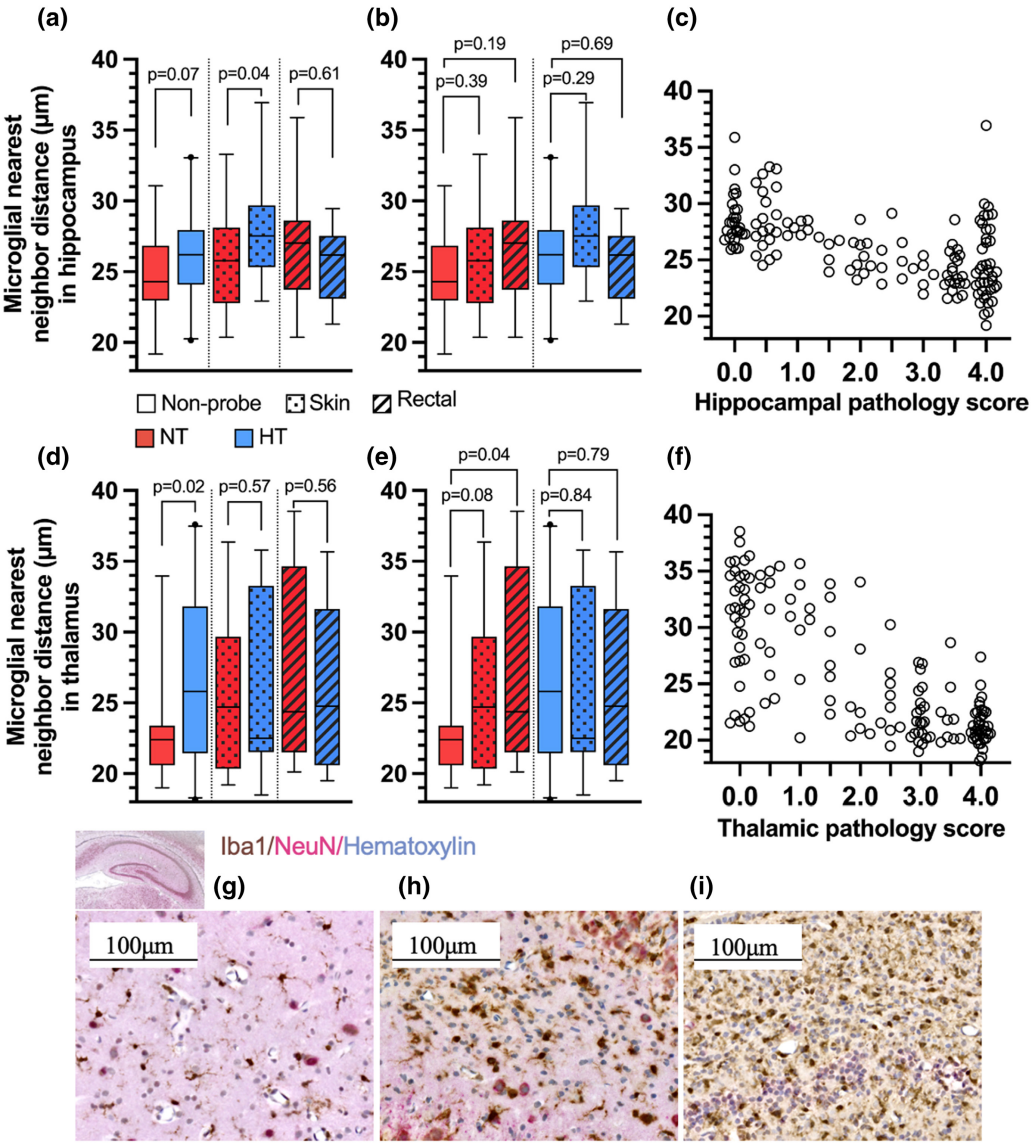
Microglial reactivity. Box plot (median, IQR, 2.5–97.5 percentile) of microglial nearest neighbor distance (NND) in the hippocampus (a–c) and thalamus (d–f) stratified by treatment (NT, HT) and probe status. NND strongly correlated with the regional pathology score in both the hippocampus (c) and thalamus (f). Representative images from the cornu ammoni in the hippocampus show microglial morphology and density at hippocampal pathology score 0.0 (g, average NND = 29.97 μm), score 2.5 (h, average NND = 25.86 μm), and score 4.0 (i, average NND = 20.17 μm). Histological images were acquired with Zeiss Axioscan Z1 (pixel resolution: 0.220 μm × 0.220 μm, objective: Plan/apochromat 20x/0.8 M27).

In the hippocampus, NND correlated with the regional hippocampal pathology score (Kendall's tau‐b − 0.44, *p* < 0.001, Figure 4). Hippocampal NND was significantly greater in the HT treatment arm in both non‐probe controls (NT: 24.3 μm; 22.9–26.8 vs HT: 26.2 μm; 24.1–27.9, *p* = 0.07) and skin probe rats (NT: 25.8 μm; 22.8–28.1 vs HT: 27.5 μm; 25.3–29.7, *p* = 0.04). There was no difference in NND among NT rectal probe rats (27.0 μm; 23.7–28.6) and HT rectal probe rats (26.4 μm; 23.2–27.9, *p* = 0.60). We detected no differences in NND among non‐probe controls, skin and rectal probe rats.

In the thalamus, NND likewise correlated with the regional thalamic pathology score (Kendall's tau‐b − 0.514, *p* < 0.001, Figure 4). Thalamic NND was significantly greater in HT non‐probe controls than NT controls (*p* = 0.019); however, no differences were detected between treatments among skin probe rats (*p* = 0.57) or rectal probe rats (*p* = 0.56). Moreover, we detected significantly greater NND in NT rectal probe rats compared to NT non‐probe controls (*p* = 0.042), whereas HT rectal probe rats were not different from HT non‐probe controls (*p* = 0.79).

### 3.5 BDNF signal intensity

**FIGURE 5:**
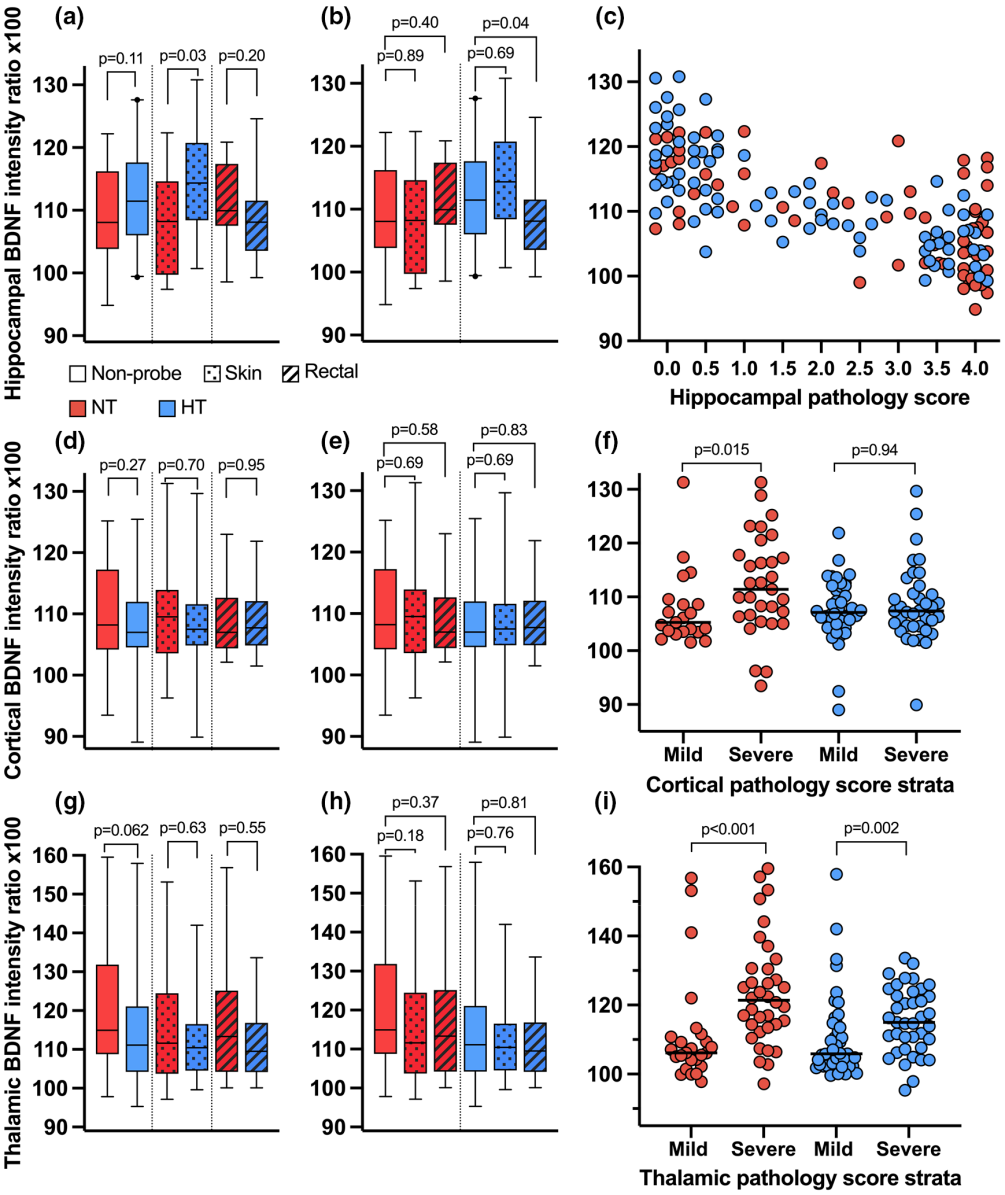
BDNF signal intensity. Mean signal intensity of brain‐derived neurotrophic factor (BDNF) in CA1‐CA4 cell layer of the left hippocampus stratified by treatment (a and b, boxplot, median, IQR, 2.5–97.5 percentile). The hippocampal BDNF signal intensity strongly correlated with hippocampal pathology score (c, scatter plot, Kendall's tau‐b − 0.56, *p* < *p* < 0.001), as hippocampus is the region most susceptible to HI injury. There were no significant differences among any of the groups. BDNF signal intensity in the cortical neurons (d and e, median, IQR, 2.5–97.5 percentile) in an area selected to avoid confounding from cell death. When stratified by treatment and mild (score 0.0–2.0) or severe (score 2.5–4.0) cortical pathology score (f, line shows median), the normothermic severe group had significantly greater BDNF signal intensity than the mild group (*p* = 0.015). In the thalamus, the BDNF signal intensity (g and h, median, IQR, 2.5–97.5 percentile) yielded comparable results. Following stratification by treatment and binary thalamic pathology score (i, line shows median), an increase in BDNF signal intensity was observed in both severe treatment groups.

The hippocampal signal intensity of BDNF correlated with the regional hippocampal pathology score (Kendall's tau‐b − 0.56, *p* < 0.001; Figure 5). Initially, we observed a stronger BDNF signal intensity in the HT treatment arm in skin probe rats (NT: 108.2; 97.4–114.6 vs. HT: 114.3; 108.4–120.7, *p* = 0.028). This finding was not significant in the non‐probe controls (NT: 108.1; 103.8–116‐2 vs. HT: 111.5; 106.0–117.6, *p* = 0.11) or rectal probe rats (NT: 109.9; 107.5–117.4 vs. HT: 107.5; 102.9–111.2, *p* = 0.20). However, following stratification by hippocampal pathology score, this difference became non‐significant in all groups.

In the cortical neurons, BDNF signal intensity was not correlated with cortical pathology score (Kendall's tau‐b 0.06, *p* = 0.31), as we deliberately selected an area not sensitive to injury in this model. Initially, no differences in signal intensity were observed between the groups (Figure 5). However, following stratification by a binary cortical pathology score (mild score: 0.0–2.0, severe score: 2.5–4.0), a significant increase in signal intensity was observed in the NT severe group irrespective of probe status (*p* = 0.015, Figure 5). The signal intensity in the HT group was unaffected by injury severity (*p* = 0.94).

In the thalamus, BDNF signal intensity correlated with thalamic pathology score (Kendall's tau‐b 0.381, *p* < 0.001). We detected no statistical differences between the groups (Figure 5). Following stratification by binary thalamic pathology score (mild score: 0.0–2.0, severe score: 2.5–4.0), an increase in BDNF signal intensity was detected in the severe group in both NT (*p* < 0.001) and HT (*p* = 0.002, Figure 5).

## 4 DISCUSSION

Unanesthetized experimental animals often require prolonged immobilization to allow for continuous physiological recordings. Likewise, infants with moderate–severe HI encephalopathy undergoing HT require some degree of restraint to facilitate fitting of the cooling equipment, mechanical ventilation and invasive lines for fluids and drugs. In the current study, we have examined the effect of restraint during a HI insult and subsequent treatment in a widely used model of neonatal HI injury in P7 rats. Our findings demonstrate a reduction in acquired brain injury in NT rats immobilized for either skin or rectal temperature monitoring, confirming, and adding on to our previous finding reported in 1996 (Thoresen, Bågenholm, et al., 1996). Injury was mitigated in the hippocampus, cortex, thalamus and basal ganglia. Neuroprotection from restraint stress was greater among the rats carrying a rectal probe. Assuming that the relatively large size and rigidity of the rectal probe causes greater discomfort/pain than the superficial skin probe, this finding suggests an added neuroprotective effect of discomfort to the restraint stress at physiological temperature (NT). In rats exposed to post‐insult HT, restraint‐associated neuroprotection was maintained in the skin probes. In contrast, HT rats carrying a rectal probe acquired significantly greater brain injury, suggesting a deleterious effect from combining cooling, stress, and discomfort.

Stress and HI injury independently modulate thermoregulation in rodents. Acute stress may induce spontaneous transient hyperthermia, as demonstrated in adult rats exposed to stressors such as rectal thermometer insertion, tail pinching and immobilization (Natarajan et al., 2015; Oka, 2018). On the other hand, HI injury induces spontaneous hypothermia in rats through a combination of physiological and behavioral thermoregulatory responses (Wood & Gonzales, 1996), which correlates with the acquired brain injury (Wood et al., 2018). It is possible that the interplay of these thermoregulatory mechanisms may contribute to the temperature‐dependent biphasic effect of restraint‐associated neuroprotection and discomfort. Because the difference in recorded temperature between each skin and rectal probe was greater during HT (1.9°C) than NT (0.8°C), we interpret this as an intact thermoregulatory response by peripheral vasoconstriction in the skin to prevent heat loss during HT.

Moreover, restraint stress, as per design, increases the levels of plasma corticosterone, the primary stress hormone in rodents (Gong et al., 2015; Jameel et al., 2014). Discomfort from the rectal probe insertion presumably exacerbates this stress response. The affinity of glucocorticoids to corticosteroid binding globulin (CGB), the primary transport protein and depot in plasma, is demonstrated to be temperature sensitive in recombinant human CGB (Chan et al., 2013). The affinity decreases non‐linearly (thus increasing the concentration of free available corticosteroid) with increasing physiological temperature, albeit with a more dramatic effect in the hyperthermic range (Chan et al., 2013). Assuming the same effect in P7 rats, we may speculate the concentration of free corticosterone in NT rats to be greater than in HT rats, which could provide a mechanistic explanation for the drastic difference in injury between NT and HT rectal probes. Corticosteroids may have neuroprotective properties (Malaeb & Stonestreet, 2014), and dexamethasone, a synthetic corticosteroid, was shown to reduce HI brain injury in P7 rats (Harding et al., 2016).

Furthermore, we hypothesized restraint to potentially alter the neuroinflammatory response. Stress and HI injury induce morphological and cellular changes in microglia in the rodent brain (Calcia et al., 2016; Hellström Erkenstam et al., 2016; Mallard et al., 2019). We examined microglial reactivity in the hippocampus, the region most susceptible to HI injury in the Vannucci model, and the thalamus, which is involved in thalamocortical pain processing. Among non‐probe controls, greater NND was observed following HT in the hippocampus and thalamus. The same finding was observed among skin probe rats, however only in the hippocampus. These findings suggest a reduced inflammatory response during HT, which is supported by the strong correlation between NND and the pathology score. Regarding differences between non‐probe controls and rectal probe rats, we report significantly reduced neuroinflammation in the thalamus in NT rectal probe rats; however, this finding was not reproduced in the hippocampus. The role of neuroinflammation in restraint‐associated neuroprotection remains unclear, and requires further studying.

Neuronal survival and synaptic plasticity is modulated by BDNF, a potential neuroprotective growth peptide (Chen et al., 2013). We measured BDNF signal intensity in the hippocampus, cortex, and thalamus as these regions contain the highest concentration of BDNF in the rat brain (Katoh‐Semba et al., 1997). Due to its susceptibility to HI injury, the hippocampal BDNF signal intensity was strongly influenced by hippocampal injury and necrotic cell remnants. We also analyzed a cortical area predominantly supplied by the anterior cerebral artery, which is usually spared from major injury. In this cortical region, BDNF intensity increased with higher cortical pathology score in the NT treatment arm, irrespective of probe status, suggesting increased protein expression from HI injury. We observed the same effect of injury severity on BDNF in the thalamus. Following HT, the cortical BDNF signal intensity was unaffected by injury severity, whereas increased BDNF expression was observed with increasing injury in the thalamus.

There are several limitations to our study. The experiments included in the analysis were conducted between 2013 to 2016, and while the laboratory, staff and protocol remained unchanged, the experiments were still susceptible to uncontrolled variables. We controlled for this by matching the non‐probe controls to each skin and rectal probe. Moreover, 34% of the litters analyzed were crossfostered by an older dam, which we recently reported to exacerbate HI injury in a mild Vannucci model (Gundersen, Menassa, et al., 2021). However, in the current study we found no significant difference between crossfostered and non‐crossfostered rats, presumably due to the longer insult duration (90 min) diminishing this effect. Another limitation of our study is the lack of quantification of stress responses such as blood levels of corticosterone. We therefore rely on the assumption that the rectal probe causes discomfort to explain the difference in injury. Lastly, immunohistochemical analysis is less accurate than comparable analytical techniques like western blot. BDNF protein expression is relatively scarce in the brain (Notaras & van den Buuse, 2020), which complicates immunohistochemical analysis. Moreover, we did not stain for the pro‐BDNF isoform, which accounts for 10% of BDNF expression (Notaras & van den Buuse, 2020) and elicits counteracting effects through induction of apoptosis (Miranda et al., 2019).

We are cautious to draw clinical implications from our findings. Restraint and discomfort during clinical care varies and may be difficult to quantify. Ventilated patients undergoing HT are usually administered sedation and analgesia throughout the cooling period (Gundersen, Chakkarapani, et al., 2021), though some restraint in the setting of intensive care is necessary. Discomfort and restraint‐related stress inflicted in the clinical setting need further studies on hormonal and physiological responses in order to document adverse or beneficial effects, though we believe experimental results such as ours are useful in highlighting potential unforeseen effects of routine clinical management.

## 5 CONCLUSION

We have conducted a meta‐analysis of 23 Vannucci experiments in the P7 rat. Our findings demonstrate reduced susceptibility to HI injury after post‐insult NT in rats immobilized to carry a skin or rectal thermometer probe. Restraint‐associated neuroprotection was greater in the rectal probes, suggesting an added effect from discomfort associated with the rectal probe. Following post‐insult HT, skin probes maintained the restraint‐associated neuroprotection. In contrast, the injury was greatly exacerbated among HT rectal probes. Our findings indicate a damaging effect from the combination of restraint, discomfort, and HT.

## AUTHOR CONTRIBUTIONS

All authors fulfill the ICMJE‐criteria. The authors confirm contribution to the manuscript as follows: study conception and study design: Julia K. Gundersen, David A. Menassa, Marianne Thoresen; data collection: Julia K. Gundersen, Hemmen Sabir, Thomas R. Wood, Damjan Osredkar, Mari Falck, Else M. Loeberg, David A. Menassa, Marianne Thoresen; analysis and interpretation of the results: Julia K. Gundersen, Hemmen Sabir, Thomas R. Wood, Damjan Osredkar, Mari Falck, Else M. Loeberg, Lars Walloe, David A. Menassa, Marianne Thoresen; draft manuscript preparation: Julia K. Gundersen, Lars Walloe, Marianne Thoresen; manuscript revision for important intellectual content: Julia K. Gundersen, Hemmen Sabir, Thomas R. Wood, Damjan Osredkar, Mari Falck, Else M. Loeberg, Lars Walloe, David A. Menassa, Marianne Thoresen. All authors have reviewed the results and approved the final version of the manuscript. The authors agree to be accountable for all aspects of the work. There are no conflicts of interest to declare.

## FUNDING INFORMATION

We are grateful for funding from The Research Council of Norway (NFR) FRIPROBIO ES492246, SPARKS UK (The Children's Medical Research Charity) 12LEO01, Forskerlinjen (NFR), University of Oslo.

## ETHICS STATEMENT

All experiments were approved by the University of Oslo’s Animal Ethics Research Committee and performed by individuals holding an approved license according to the Animal act 1986, FOTS ID: 4344.
